# Monkeypox virus (MPXV): A Brief account of global spread, epidemiology, virology, clinical features, pathogenesis, and therapeutic interventions

**DOI:** 10.1016/j.imj.2023.11.001

**Published:** 2023-11-05

**Authors:** Md Aminul Islam, Jubayer Mumin, Md Masudul Haque, Md. Azizul Haque, Ahrar Khan, Prosun Bhattacharya, Md Atiqul Haque

**Affiliations:** a Advanced Molecular Lab, Department of Microbiology, President Abdul Hamid Medical College, Karimganj 2310, Bangladesh; b COVID-19 Diagnostic Lab, Department of Microbiology, Noakhali Science and Technology University, Noakhali 3814, Bangladesh; c Department of Global Public Health, Karolinska Institutet, SE-171 77 Stockholm, Sweden; d Department of Public Health, North South University, Dhaka 1229, Bangladesh; e Department of Biochemistry and Molecular Biology, Faculty of Agriculture, Hajee Mohammad Danesh Science and Technology University, Dinajpur 5200, Bangladesh; f Shandong Vocational Animal Science and Veterinary College, Weifang 261061, China; g COVID-19 Research @KTH, Department of Sustainable Development, Environmental Science and Engineering, KTH Royal Institute of Technology, SE-100 44 Stockholm, Sweden; h Key Laboratory of Animal Epidemiology and Zoonoses of Ministry of Agriculture, College of Veterinary Medicine, China Agricultural University, Beijing 100019, China; i Department of Microbiology, Faculty of Veterinary and Animal Science, Hajee Mohammad Danesh Science and Technology University, Dinajpur 5200, Bangladesh

**Keywords:** Monkeypox (MPX), Monkeypox virus (MPXV), Epidemiology, Zoonotic diseases, Re-emerging viruses, Genomic dissemination, Vaccination and Treatment

## Abstract

The largest monkeypox virus (MPXV) outbreak of the 21st century occurred in 2022, which caused epidemics in many countries. According to WHO, physical contact with infected persons, contaminated surfaces, or affected animals might be a source of this virus transmission. A febrile sickness including few symptoms found in MPX disease. Skin rash, lesions, fever, headache, fatigue, and muscle aches symptoms were observed commonly for this disease. Animal and in vitro, studies have shown that the antiviral medications cidofovir and brincidofovir are effective against MPXV. The first-generation vaccinia virus vaccine was developed in 1960, and it helped to protect against MPXV with its side effects. A second-generation vaccination with limitations was launched in 2000. However, the CDC advised vaccinations for risk groups in endemic countries, including positive patients and hospital employees. The JYNNEOS vaccine, administered in 2 doses, also provides protection from MPX. This article presents concisely the most recent findings regarding epidemiology, genomic transmission, signs and symptoms, pathogenesis, diagnosis, and therapeutic interventions for MPXV, which may be helpful to researchers and practitioners. WHO declared that MPX was no longer a global health emergency due to its declining case rate, and a number of countries have reported new incidences. Further research-based investigations must be carried out based on the 2022 outbreak.

## Highlights

• The largest 2022 Monkeypox (MPX) outbreak has changed its nature rather than earlier including clinical signs symptoms, severity, and epidemiology.
• MPX occurrences are declining; however, awareness must be raised on this contagious disease to stop further outbreaks.
• More research-based studies need to be carried out to find our virus morphology, its genomics, mode of transmission, reservoirs, and therapeutic treatment.

## Graphical Abstract

**Figure:**
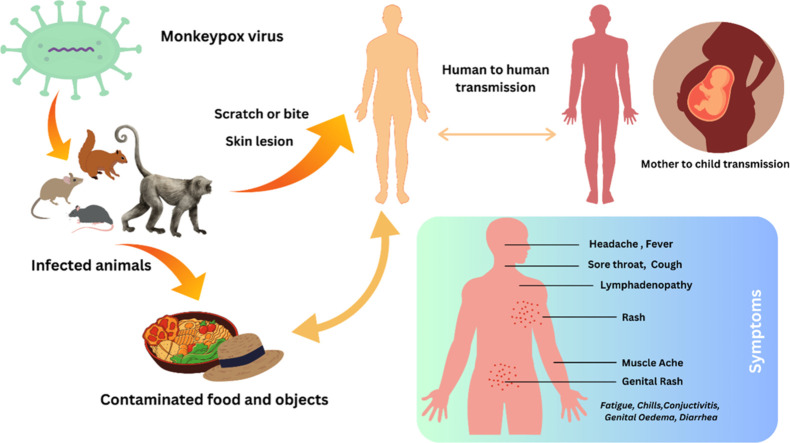

## 1 Introduction

The emergence of the COVID-19 pandemic has raised public awareness of zoonoses and the global hazards posed by new emerging pathogens [1]. The COVID-19 pandemic has prompted a surge in investment in wildlife virology worldwide, and new surveillance programs are expected to uncover hundreds of novel viruses that could 1 day threaten human health [2]. In 2021, experts predict there will be 887 zoonotic viruses, which primarily harm animals, with the potential to cause a global pandemic [3]. Both the monkeypox virus (MPXV) and the cowpox virus (CPXV) belong to the poxviridae family and are recorded as the top 50 most dangerous viruses [4]. MPXV is an enveloped double-stranded DNA (dsDNA) virus, that belongs to the *Orthopoxvirus* (OPXV) genus similar to that of vaccinia, variola, and cowpox virus (CPXV). Although the virus was initially found in Africa, it has since spread to new areas that have no ties to West or Central Africa. According to WHO MPX data, a total of 89,752 positive cases with 157 death cases were recorded. The majority of reported cases have involved homosexual and bisexual men between the ages of 20 and 50; nevertheless, it is unclear whether sexual practices are the primary factors in its transmission [5]. The important way of transmission of MPX is sexual contact with an infected individual [[6], [7]]. Most of these positive cases related to traveling history who previously traveled from Europe and North America or contact with positive patients. According to the MPX risk assessment conducted by the World Health Organization, various regions of the United States are categorized as high-risk groups. However, no sufficient data supports the respiratory transmission route for this virus [8]. Poxviruses contain lower lipid content in the envelope, so they are more resistant to environmental conditions. A total of 179 samples were tested from MPX-positive patients' rooms with high-efficiency particulate air filters (HEPA), where 1 week of contamination was found that declined after 3 weeks [9]. It is also presumed that the MPX virus enters human body through different channels such as broken skin, respiratory tract, or mucosal surfaces (e.g. oral, pharyngeal, ocular, genital, anorectal). In 1958, MPXV was identified in a vesicular disease outbreak in captive African monkeys shipped to Copenhagen, Denmark, from Central and West Africa [10]. Rats, mice, prairie dogs, squirrels, and possibly even humans are all considered natural reservoirs in Africa. In addition, the Gambian pouched rat (*Cricetomys gambianus*), the dormice (*Graphiurus spp.*), and the African squirrels (*Heliosciurus, and Funisciurus*) were all found to be carriers of this virus. Although the 2022 outbreak spread from human to human, these animals earlier also worked as a transmission source [11].

After the first identification of MPX in monkeys in 1958 from Denmark, several cases were recorded. In 1970, the first incidence in humans was documented. Initially, MPX cases were familiar in the African countries where the Democratic Republic of the Congo (DRC), Cameroon, Nigeria, Gabon, Central African Republic, South Sudan, Sierra Leone, and Liberi were notably documented [12]. In 2022, the number of positive individuals increased in all 5 continents. MPX is classified as an emerging disease by the World Health Organization (WHO) and the Centres for Disease Control and Prevention (CDC) due to its high infectivity and rapid dissemination [13]. However, the characteristics of the 2022 MPX outbreak have changed its nature, including clinical manifestation, severity, and epidemiology. The US Food and Drug Administration (FDA) and the European Medicines Agency (EMA) have approved the use of the antiviral drugs Tecovirimat and Cidofovir for treating MPX infection [14]. One vaccination has been approved for use against MPX in the European Union (EU) since July 22, 2022. The Jynneos Smallpox and Monkeypox Vaccine, Live, Non-Replicating, was given FDA approval in September 2019. However, 3 vaccines, Imvanex (Jynneos or Imvamune), ACAM2000, and LC16m8, have enhanced efficacy against Orthopoxvirus infections while adequately stimulating antibody production in severely immunocompromised patients [15].

On May 10, 2023, the WHO declared that a global outbreak of MPX did not constitute a public health emergency on a global scale [16]. However, further MPX waves and outbreaks might appear, and the committee suggested being concerned about new, significant cases. On June 22, 2023, the first reported case of Mpx in Israel was a 50-year-old Portuguese tourist [17]. Despite receiving the vaccination, the Ministry of Health confirmed that this individual had contact with infected people. Following public health regulations and MPX disease prevention and control strategies is vital. Health worker personnel related to MPX patients should follow adequate precautions, hygiene, and maintain personal protective equipment (PPE) when handling patients, and educational programs may be useful to increase public awareness. MPX-positive cases must be isolated in a separate room with proper care and treatment. In the hotspot locations, serological surveillance, contact-based surveillance, and surveillance based on wastewater can all be started for confirmed cases [18] Mass vaccination campaigns may be implemented in the outbreak areas to reduce cases. This review article provides a concise overview of MPX viral history, epidemiology, genomic dissemination, symptoms and signs, pathophysiology, diagnosis, and therapeutic interventions, as it may benefit bench researchers and practitioners.

## 2 Historical background

In 1958, a vesicular rash resembling smallpox (SPX) was observed in African cynomolgus monkeys that had been transported to Copenhagen, Denmark, for research endeavors. After this event, this rash was named “MPX” [4]. From 1981 to 1986, DRC reported over 300 confirmed instances of MPX in humans. Since its reappearance in 1996, another 400 cases have been recorded, with over 80% of patients related to human-to-human transmission. At least 47 cases were confirmed during the 2003 outbreak in the United States 6 Midwestern states—Illinois, Indiana, Kansas, Missouri, Ohio, and Wisconsin [19]. It is also found that no deaths were reported for MPX in Central or West Africa between 1970 and 2019 [7]. An epidemic was documented in 2005 in the Democratic Republic of the Congo. More than 200 human cases of MPX were reported in Nigeria between 2017 and 2018, with positive cases confirmed in the United Kingdom (UK) in 2018 [20]. The case fatality rate (CFR) observed during the monkeypox outbreak in Nigeria from 2017 to 2018 was approximately 6%. From 2018 to 2021, no instances of fatalities were reported inside the geographical boundaries of the United Kingdom.

Several MPX outbreaks were also observed in the different countries of Central and West Africa. However, maximum positive cases were reported from outside Africa, linked with infected travelers directly or indirectly. The first MPX report in the USA appeared in 2003. In 1 shipment, there were 800 animals from 9 species carried for laboratory experiments. Some of the MPX-affected animals were housed at the facilities of an Illinois animal trader alongside prairie dogs after they were imported to the United States. Before they showed any sickness symptoms, these prairie dogs were sold as pets. Prairie dogs living close to imported rodent species from Ghana, West Africa, had this outbreak. As a result, the prairie dogs transmitted MPXV to about 40 human cases, later confirmed in laboratories as human MPX [21]. Rodents, carnivores, insectivores, nonhuman primates, and other mammals worldwide have been documented to acquire MPXVor by experimental means. Although African squirrels and/or other rodents are the strongest contenders, these findings have made it more challenging to identify the natural host. Therefore, it is unclear which mammal(s) serves as the virus reservoir(s).

In the Democratic Republic of the Congo, a young boy, the only unvaccinated member of his family, reported the first human case of monkeypox in 1970 [22]. The next decade witnessed widespread reports of monkeypox among youngsters across several countries in Central and Western Africa, including the DRC, with a mortality rate of 11% among those who hadn't been vaccinated against smallpox [21]. On April 4, 1971, a little girl from the south-eastern Nigerian town of Ihie-Imuduru came down with a high fever and vesiculopustular skin lesions [23]. Her 24-year-old mother also developed a fever and a rash. This young girl was identified as Nigeria's first confirmed case of monkeypox following laboratory testing, and her mother's sickness was reported as the first suspected case of human-to-human transmission of monkeypox. In the 2017 MPX outbreak reported in Nigeria, most infected individuals were young adult men (69%). Since 2017, Nigeria has observed an increase in MPX cases, with over 500 instances reported. These cases have been linked to travel from the UK, Israel, Singapore, and the United States [24]. The first report in Europe was found in the UK on May 6, while the first case in the US was detected in Boston, Massachusetts, on May 17.

## 3 Epidemiology

The 2022 outbreak exhibits several atypical characteristics in terms of positive cases. As of September 04, 2023, there were 89,752 confirmed cases of MPVX infection, resulting in 157 fatalities across 113 countries, making it a significant focus due to its infectiousness and role in numerous human health complications (Table 1). Before 1970, the MPXV was only known to infect nonhuman primates and had also been found in African rodents [25]. From 1971 to 2017, the MPXV was predominant in the West and Central African regions, where the different animals served as intermediary hosts. In 2022, MPX suddenly extended to nonendemic locations beyond the African continent, causing a multicountry outbreak with high fatalities. The sudden rise in viral exposure and subsequent infections in several continents predominate scientific focus since it was primarily contained in Africa since its inception [25]. The analysis of WHO 2022, commonly abbreviated as MPX Outbreak: Global trends, reveals that the number of new positive to MPX cases increased by 76% (*n* = 280 cases) from January 30 to February 5 compared to the previous week (*n* = 159 cases), with the highest incidence occurring in the Region of the Americas (83%) (Table 1) (WHO, 2022) [26].

**Table 1:** Number of confirmed cases, deaths and case fatality rate of MPX outbreak from January 2022 to July 2023 (WHO 2022) [26].

Regions	Confirmed cases	Deaths	Case fatality rate (%)	Week-old cases (Last week)	Week-old cases (Preceding week)	Cases (%) weekly
America	59,638	120	0. 20%	84	67	25%
Europe	25,935	7	0.02%	23	22	5%
Africa	1802	21	1.16%	0	67	−
Western Pacific	891	0	0%	70	79	−11%
Eastern Mediterranean	90	1	1.11%	0	0	
South-East Asia	147	1	0.68%	63	33	91%
Total	88,503	150	0.16%	240	268	−10%

According to WHO data, the highest concentration of cases occurred in people aged 27–41 (3780/4777; 79.1%), the vast majority of patients were male (81,150/84,258; 96.3%), 52.5% were HIV-positive (17,827/33,954), and 96% of men identified had a history of sexual activity with other men. According to the latest weekly report (July 3–July 9, 2023) data, global MPX cases increased by 9.2% (*n* = 83) between weeks 35 (August 28–September 3) and 34 (August 21–August 27). In the first 4 weeks of 2023, 3561% of MPX cases were in the Western Pacific Region, while 28.6% were in Africa. United States of America (30,564), Brazil (10,967), Spain (7565), France (4150), Colombia (4090), Mexico (4055), Peru (3812), the UK (3777), Germany (3696), and Canada (1,496) are the top ten nations affected (Supplemental Table S1). It is found that MPXV clades affect the patients’ mortality rate. Clade IIa (which corresponds MPX to one of the “West Africa Clades”) has lower fatality rates than Clade I (formerly known as the “Congo Basin Clade”), which is more clinically severe. At least 47 recorded human cases of MPX between 1970 and 1979 in West and Central Africa had 49% severe disease, and 17% of the patients passed away from acute sickness. The overall death rate for children who had not had a smallpox vaccination in the central parts of the DRC between 1981 and 1986 was as high as 9.8%. With clade IIb disease, mortality is a serious worry in pediatric clade I infections (clade I, mortality of about 10%, clades IIa, 1%–3%, and clade IIb, 0.05%), is incredibly rare.

**Fig. 1:**
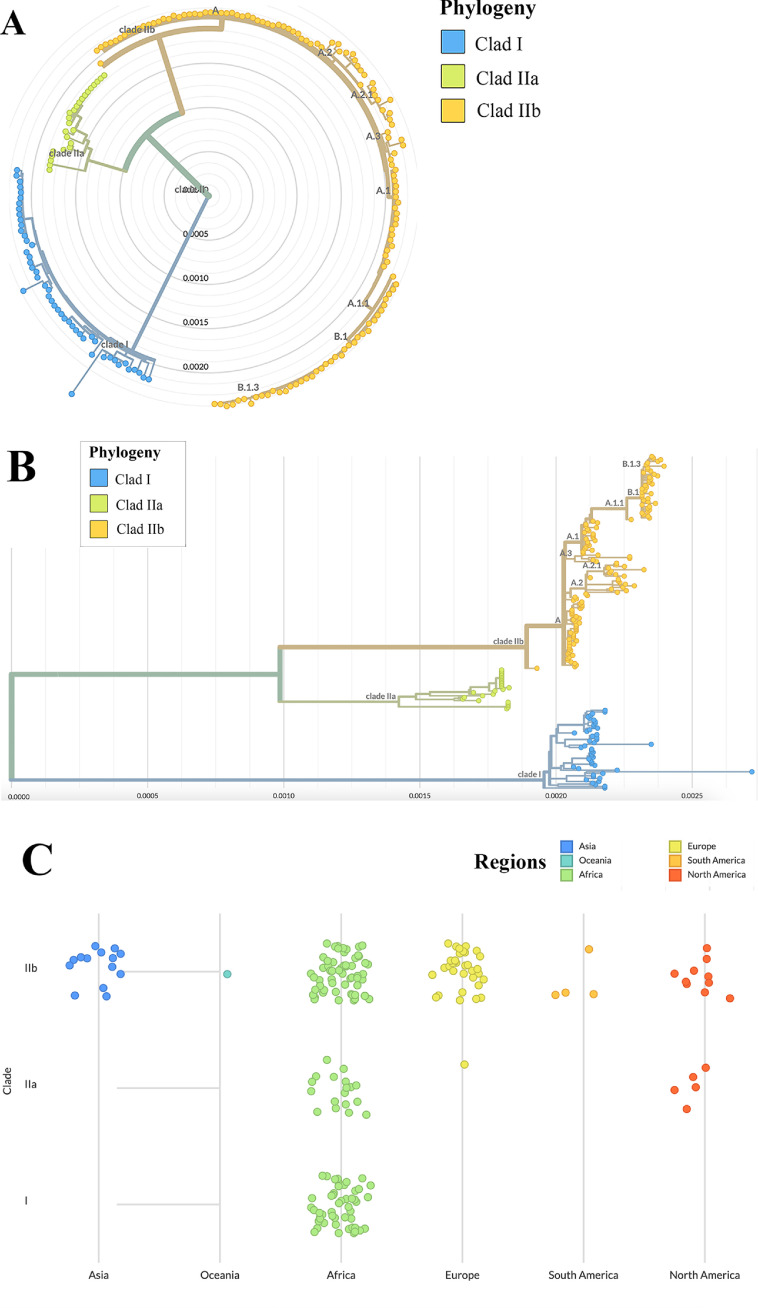
Transmission of genomes of MPX worldwide. (A) Phylogenetic lineage of MPX; (B) Distribution of MPX clades; (C) Continental transmission of MPX genome (Genomic epidemiology of MPXV-https://nextstrain.org/mpox/all-clades).

Since May of 2022, the number of reported cases of MPXV has skyrocketed over the globe. Although it has decreased, new cases are still counting in the few areas of the world. MPXV, a DNA virus with a genome size of 197 kbp and more than 190 open-reading frames (ORFs), forms mainly 2 clades in phylogeny analysis: The central African clade (Clade I) and the West African clade Clade II (IIa and IIb) [27]. Fig. 1 depicts the global genomic transmission of MPX [28]. After reviewing the timeline of the phylogeny in Nextstrain, B.1 variant was first screened and deposited from the United Kingdom in April 2022. It was later identified in other European countries and in North America, namely the USA, indicating a global transmission route. However, lineage B.1, B.1.1, and B.1.2 were first identified in Germany, whereas, lineage B.1.17 originated in the USA. In contrast, B.1.16 lineage was the first to be sequenced and deposited from Columbia and it contained 62 mutations in the genome when compared to the reference sequence. Strain B.1.10 which originated in Spain, was also frequently found in Colombia.

During the early stages of the 2022 outbreak, a study of clinical samples of MPVX by phylogenetic analysis indicated a significant genetic similarity among these samples, thereby classifying them under the clade IIb. The present study has shown evidence suggesting a common source for the ongoing epidemic [24]. A proposal was put forth to designate clade IIb as hMPXV1 in order to emphasize its status as a human virus, distinct from other largely zoonotic MPXV strains. This distinction is warranted due to the ongoing human-to-human transmission observed during the 2022 outbreak [25]. Based on the study conducted, it has been determined that all the identified hMPXV1 sequences associated with this epidemic are attributed to a solitary lineage (B.1), which can be traced back to its predecessor A.1.1. The A.1.1 lineage can be traced back to the A.1 lineage, which was responsible for a substantial outbreak in Nigeria from 2017 to 2018. Furthermore, lineage B.1, which is the current lineage, has a divergence of approximately 50 single nucleotide polymorphisms (SNPs) from lineage A.1, the lineage linked to the Nigerian outbreak in 2017. The genomic analysis of hMPXV1 sequences from the current outbreak has identified a mutational bias towards the substitutions GA > AA (26 mutations) and TC > TT (15 mutations) [27]. Therefore, several transmission routes may have existed for spreading different strains of the human MPXV (Fig. 1). With a fatality rate of 10.6% compared with the 3.6% for Clade I, Clade IIa, and IIb are found to be more virulent and deadly and responsible for the recent outbreak [29]. Lineage A.1 of MPX contains unique mutations in C23564T, C83326T, and G148412A belonging to clade IIb, formerly the West African clade. According to Nextstrain data, the majority of A.1 lineage members were centered in Africa with less than 5% detected and sequenced in Singapore and Europe, namely the UK [19]. North America, namely the USA and Canada, as well as European countries (Spain, Portugal, Canada, Germany, Belgium, France, and others), were infected mostly by B.1, B.1.1, B.1.1.2, and B.1.3 strains belonging to West African variants [30]. B.1 Strain is associated with 46 and 24 synonymous and nonsynonymous single nucleotide polymorphisms (SNPs), crucial for the lineage's recent upheaval [31].

Moreover, whether the A3 subfamily of APOBEC, which is intricately linked to the deamination of cytidines into uridines, is gone under gene editing is also circulated for further discussion [30]. Genetic analysis suggests that APOBEC-mediated editing is accountable for the very high number of mutations found in hMPXV1 genomes. B.1 was found to be the most prevalent variation after studying the mutation profiles of 1624 hMPXV1 sequences. Most MPX mutations were C-to-T and G-to-A transitions, and they typically occurred in a sequence context (5′-TC-3′) indicative of the preferences of several human APOBEC3 enzymes [32]. Although researchers found no evidence of a transcriptional imbalance, Forni et al. (2023) noticed a bias toward mutation clustering in highly expressed viral genes [33]. According to an article, glycoprotein-based receptor B21R, which harbors 3 nonsynonymous SNPs in M17411, D209N, and P722S is linked to viral infectivity of B.1, responsible for the majority of infections around the globe. Strain B.1.17, a subset of B.1 also made up the significant proportion of infection in the USA while B.1.14, B.1.15, and B.1.16 contribute to some infections in USA and Germany [27].

The question of whether or not droplets can spread monkeypox remains open. Due to the short half-life of respiratory droplets, direct contact between people requires more time for transmission. Notably, there has been no confirmed case of this disease being transmitted by air travel [34]. One observational study was conducted for air and surface samples across Monkeypox hospitals. Using RT-qPCR, 93% of surface samples (*n* = 60) and 25% of air samples (*n* = 20) were detected as positive.  PPE was also observed following clinical interaction. This study showed that the MPXV was present in the air and the surface [35]. Another study used aerosol suspensions to test the virus's vulnerability from inside a circulating chamber in a class III biosafety cabinet. This virus was identified after 90-hour using viral culture and RT-PCR found a correlation between viral load and both body surface area and saliva [[36], [37]]. This report also recommends additional investigation into the 2022 MPX outbreak's respiratory route of infection.

The first MPX case was confirmed in Islamabad, Pakistan, on April 21, 2023. The patient, a Pakistani guy of 25 years old, had returned from Saudi Arabia and was now showing signs of illness. The patient was admitted to hospital isolation to undergo diagnostic testing due to the severity of his illness. Health officials released no further information about the guy, but people with confirmed cases of monkeypox were screened for contacts and encouraged to self-quarantine.

## 4 Clinical features

MPX is a viral infection that can be transmitted to humans; its clinical signs resemble those of smallpox but are milder. However, the severity may vary widely from patient to patient and from condition to condition. Upon exposure and infection, an incubation stage lasts about 4–21 days [38]. This is followed by a prodrome step that lasts about 1–3 days, wherein patients begin to experience general viral infection signs and symptoms, and usually, the disease clears itself within 2–4 weeks [21]. Patients with the MPXV outbreak in 2022 have been seen to have high fever, rash, asthenia, fatigue, headache, enlarged lymph nodes, sore throat, chills nausea, breathlessness, dysphagia, angina, myalgia, genital necrosis, weaken digestive tracts, inguinal lymphadenopathy, exanthema, conjunctivitis, eye damage resulting from corneal infection, diarrhea, and vomiting resulting in dehydration, encephalitis, tonsillitis, pharyngitis and, uncommonly, bronchopneumonia [39]. The first phase of MPX infection is observed anywhere from 7 days to 17 days (with an average of 12 days) after the first exposure. Although there are no outward symptoms during this time, the MPXV virus is actively replicating and spreading throughout the host. Prodromal phase symptoms typically linger for many weeks, making it a lengthier time span. The nature of the illness and the degree to which patients are affected may differ [38].

According to clinical data-based evidence, men are more likely to be infected (99%), have homosexual tendencies (94%), and make up a substantial portion of infections in the USA across people of color [40]. The overall death rate varies based on patient age, viral clade, and epidemic location. The majority of recorded deaths have been in immunocompromised patients, young adults, and children, with a rate of 1%–10% and higher fatality, stronger rash, and more severe clinical manifestations in unvaccinated patients [41]. It is estimated that the overall MPX case fatality rate was 8.7%, with a substantial variation between clades: 10.6% (95% CI: 8.4%–13.3%) in Central Africa vs. 3.6% (95% CI: 1.7%–6.8%) in West Africa [19]. The majority of male patients were between the ages of 27 and 41 (780/4777; 79.1%), and the majority of male patients were also HIV-positive (48.4%; 17,558/36,308) and had a history of having sex with other men [26]. The virus is also associated with severe complications during the pregnancy period among women, including premature miscarriage, congenital infection, skin inflammation in the fetus, weight loss, and maternal death [42]. It has been observed that MPX can be passed from mother to child, even though there is very little clinical data regarding MPX during pregnancy [43]. Numerous unwanted side effects, miscarriages, and stillbirths have been linked to MPX. Positive cases in neonates and preterm births have also been documented [44].

**Fig. 2:**
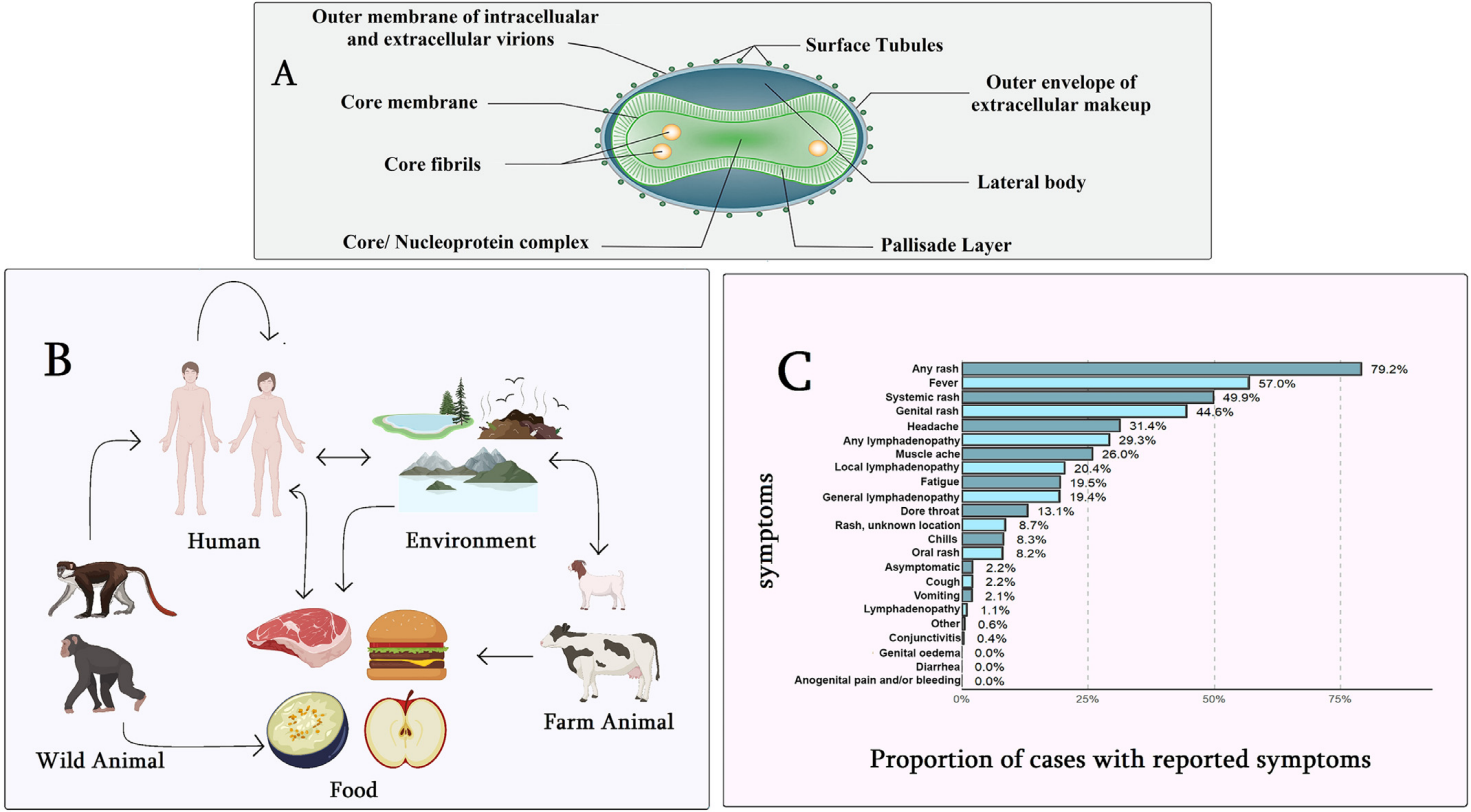
Comprehensive scenario of Monkeypox. (A) Virology (B) Transmission cycle (C) Symptoms [32].

It is observed that the ongoing outbreak is a little more distinct than the previous one. It has changed its infectivity to mild and spread in a limited part of the human body. It is reported that 94% of MPX patients with anogenital lesions and 56% of those with lymphadenopathy had a fever [45]. Five people (or 9%) were admitted to the hospital, and 13 people (or 24%) were living with HIV; all were on antiretroviral medication (ART) and had normal CD4 cell counts; 11 had undetectable HIV RNA, and the rest had just started ART [45]. Almost 86% of the patients reported clinical fever, 62% swollen lymph nodes, 58% and muscle aches and pain (32%) (Fig. 2). In contrast with previous reports suggesting that such symptoms precede skin lesions, the British researchers found that 38% of patients developed lesions prior to systemic illness and 14% only developed lesions. All 197 patients in a single descriptive MPX case study exhibited skin lesions, with 56.3% located in the genital area and 41.6% in the perineal region, indicating that this outbreak was mostly a skin disease. The most prevalent symptoms reported by more than eighty percent of patients who reported sickness back were fever, lymphadenopathy, and myalgia [46].

In contrast to earlier outbreaks of MPX, the majority of cases in the 2022 epidemics have exhibited minor signs of the disease. However, it is important to note that MPXV has the potential to induce serious illness in specific demographic subsets, such as small children, pregnant women, and those with compromised immune systems. Among the cases in which individuals reported experiencing at least one symptom, the prevailing symptom is the presence of a rash, which is recorded in 90% of cases with at least one documented symptom. It should be noted that the task of selecting accurate denominators for symptomatology is challenging due to several factors. These factors include a general absence of negative reporting and variations in symptom classifications across different countries' reporting systems.

## 5 Diagnosis

It is difficult to distinguish morphologically between MPX and SPX, as both are almost similar. The incubation time for both monkeypox and smallpox is between 4 and 21 days, and the symptoms are very similar [47]. A laboratory-confirmed diagnosis of MPXV is essential for detecting MPVX infection based on clinical symptoms; therefore, healthcare professionals clinically test for MPX with fluid samples swabbed from the rash [48]. It is difficult to differentiate between monkeypox and smallpox since both diseases exhibit nearly identical signs and symptoms, except for vesiculopustular rash, lymphadenopathy, and crop-like, less centrifugally dispersed lesions. Further, different positive samples showed MPXV genes from surface lesions/ rash/ exudates /rusts using skin swabs, which could be screened by PCR, RT-PCR, cell culture, and whole genome sequencing [14].

The patient's saliva, urine, stool, and semen could also be tested for viral detection, containing viral genetic materials. Swabs, scabs, and aspirated lesion fluid should be used for PCR rather than blood due to the short duration of monkeypox viremia. Swab samples taken in a fluid medium from the rash are the most often used samples. PCR, RT-PCR, cell culture, and whole genome sequencing can be used to screen the remaining samples [49]. Thus, an organized laboratory with skilled persons requires to screen MPXV DNA. Serologic cross-reactivity across orthopoxviruses is a substantial obstacle to the epidemiologic characterization of disease outbreaks and laboratory identification of specific orthopoxvirus infections. Rapid antigen and antibody tests are also preferable with low specificity rather than other tests [50]. As the outbreak of this new zoonosis continues, it is critical to expand the laboratory network's global reach, increase laboratory capacity, and better the ability to report foreign specimens [51].

Although RT-qPCR is counted as a gold standard tool, this process is costly and needs an expert person with laboratory facilities [18]. Low and middle-income countries need a cheap diagnostic solution. Point-of-care (POC) is easier and easy to reach patients in critical countries [52]. LAMP or loop-mediated isothermal amplification is one kind of isothermal procedure using a designed primer and a probe can be used for MPX. It is a quick, low-cost, simple, and powerful approach for rapid nucleic acid amplification at a single and isothermal temperature (for example, 60°C–65°C) [53]. This assay can be conducted without the use of costly instruments. Moreover, the process requires just a few minutes to complete.

In addition, tests based on CRISPR-Cas systems can also be employed for quick diagnostic testing at the point-of-care by employing isothermal amplification methods. SHERLOCK activates the guide RNA-Cas13a complex after target sequence binding through isothermal amplification and cleavage [53]. It adjacently cleaves nearby reporter RNA coupled to a quenched fluorophore to identify pathogens. DETECTR CRISPR guide RNA-Cas12a complexes to activate upon target single-stranded or dsDNA binding. Cas12a randomly cleaves single-stranded DNA attached to a fluorescent reporter or lateral flow assay (LFA) [41].

One research group used a nanoparticle and multiple cross-displacement amplification device to test MPX and isolate variants [54]. MPXV-MCDA-LFB applied 2 primer groups focusing on the D41L and ATI genes of Central and West African MPXV isolates, using 64°C for 30 minutes. Penn State researchers created the Nanoparticle-Based Rapid Test Penn State researchers developed the first rapid monkeypox nanoparticle-based diagnostic using a molecular sensor [55].

NAAT (nucleic acid amplification tests) are used for detecting MPX to target DNA polymerase E9L, an Envelope protein (B67). Polymerase Chain Reaction or PCR can be performed from lesion samples. WHO suggested using the surface of patient lesions with swab sticks, exudates, or crusts [56]. In addition, blood, semen, urine, genital, and rectal swabs also can be collected for MPX diagnosis. Alternative to NAAT test Immunological techniques such as ELISA (enzyme-linked immunosorbent assay) may be used for immunoglobulin IgG and IgM antibody detection [51]. However, immunohistochemical staining, electron microscopy, and serum tests can also be used to detect this virus. Cell culture-based tests cannot be performed for routine tests due to time and cost. Although diagnostic procedures have advanced, they are not without their flaws. Serologic cross-reactivity amongst orthopoxvirus is just one example. Even though their specificity is poor, rapid antigen and antibody tests are nonetheless often employed. The collection, storage, transportation, processing, interpretation, and reporting of laboratory results all require better infrastructure [57].

## 6 Pathogenesis

Monkeypox is highly contagious and can spread from person to human or animal to human through intimate contact, which is the first step in the virus's pathogenicity and pathogenesis. The first step of viral pathogenesis in humans is the entry of virus particles into host cells. Extensive research has been conducted on the interaction between the virus and the host cell's response, revealing notable genetic variability in the genes encoding Clade I and II. This finding contributes to elucidating the aforementioned variations in pathogenicity exhibited by these entities. The genomes of monkeypox comprise multiple genes that encode host-response modifier (HRM) proteins. These proteins, namely poxviral inhibitors of complement enzymes (PICEs) and the MOPICE protein, are recognized as virulence factors of monkeypox. The MOPICE protein has been documented as a contributing factor to the increased pathogenicity observed in Clade I, while it is absent in Clade II. The introduction of MPXV into the human body has been envisioned via several different routes, including the respiratory (such as oropharyngeal nasopharyngeal), intradermal, or sexual routes [58]. This virus can spread from person to person in a number of ways, including by direct contact with a virus-positive person's rash, lesion, or respiratory droplets. Indirect transmission of this virus from contaminated objects is also possible. The virus multiplies at the inoculation site and spreads immediately to the local lymph nodes and certainly in blood to the spleen, tonsils, and bone marrow, causing primary viremia and transfer to tertiary organs like skin and testes [39]. In vivo studies confirmed that MPXV replicates in lymphoid tissues in the neck and throat, and after primary lymphatic spread, it infects the spleen and liver [27]. When skin-based blood cells like macrophages, dendrites, and Langerhans cells become infected, the resulting inflammation affects skin cells including fibroblasts and keratinocytes [59]. It has also been noted that some cases of MPX only cause vaginal lesions, which may point to a preferred MPXV tropism in the testes as a potential site for latent MPXV infection. Fusion and macropinocytosis are 2 ways that MPXV can enter or invade the host cell. The entry and release of MPXV are complicated by the coexistence of 2 distinct viral forms, internal mature virus (IMV) and extracellular enveloped virus (EEV), which are encased by diverse different lipid membranes and also exhibit distinctive surface proteins [60].

## 7 Pharmacological measures for prevention and treatment

Historically, MPX immunization with vaccinia virus (another orthopoxvirus) provided 85% protection. However, following the eradication of smallpox (SPX) in 1980, routine vaccination against orthopoxvirus was no longer mandatory [19]. As MPX and SPX viruses are genetically related and have minimal polymorphic antigens on their surfaces, cross-reactive antibodies can protect against MPXV infection [15]. Bavarian Nordic's tradename JYNNEOS is a Live, Non-Replicating Virus Vaccine, and the Centers for Disease Control and Prevention (CDC) has qualified it and another vaccine, ACAM2000, for use in the United States to prevent MPX [61]. Developed from the vaccinia Ankara-Bavarian Nordic (MVA-BN strain), this vaccine is effective against smallpox and monkeypox in people aged 18 and over. JYNNEOS, a smallpox vaccine administered in 2 doses, has been licensed for use in both children and adults [62]. Suppose you are homosexual, bisexual, or multisexual and have had sex with males. In that case, you can get vaccinated against mumps if you are at risk of coming into contact with an infected person or if your sex partner has tested positive for the virus [63].

The proposed therapeutics used for MPX include antiviral drugs, such as astecovirimat, cidofovir, and brincidofovir approved by FDA and EMA, along with 3 recommended vaccines (Imvanex or ACAM2000 or LC16m8) [64]. Prior vaccination may also reduce or prevent monkeypox due to its long incubation period. Imvanex is an attenuated, nonreplicating, SPX vaccine made by the Bavarian Nordic Co., Denmark, and commercialized as Imvanex (Europe), Jynneos (USA), or Imvamune (Canada) that is effective at minimizing the risk of acquiring MPX infection and requires only 2 doses subcutaneously [65]. ACAM2000, a second-generation vaccinia virus vaccine developed from a plaque-purified SPX strain, is provided in a single percutaneous dosage with a booster dose every 3 years or at least every 10 years by Sanofi Pasteur Biologics Co., USA. Conversely, the US CDC does not suggest it for adults with inherited or acquired immune deficiency diseases (particularly AIDS), skin problems like atopic dermatitis/eczema, cardiac disorders, infants under 12 months old, or pregnant women [66].

The third vaccine, LC16m8, is an attenuated SPX vaccine developed from the Lister vaccinia strain produced by KM Biologics in Japan for use in healthy persons against biological terrorism. It was developed to lack the B5R envelope protein gene of the vaccinia virus to attenuate its neurotoxicity. It produced neutralizing antibodies to vaccinia, MPX, and variola major and widespread T-cell responses [15]. The recommendation on behalf of the CDC to apply Tecovirimat as a potential therapeutic intruder in pregnant women was justified in the sense that animal studies concluded no significant adverse effects would be observable in the case of the fetus. Cidofovir blocks viral DNA polymerase-mediated DNA synthesis, whereas, brincidodofovir is an upgraded form of cidofovir with a better renoprotective profile when administered orally, promoting the inhibition of viral DNA synthesis [38]. Nucleosides can be categorized into acyclic nucleoside phosphates and liquid cidofovir conjugates. The future directives when coming to therapeutic strategies of MPX are promising. The genomic similarity of MPX is 96.3% with other pox viruses [67]. There is insufficient data to support tecovirimat's effectiveness in treating human monkeypox patients, but a human clinical trial revealed it to be safe and acceptable, per the CDC report [68].

## 8 Key measures to prevent future monkeypox outbreaks

In order to swiftly detect cases of monkeypox and prevent its spread, it is necessary to develop and distribute effective diagnostic assays worldwide. Unnoticed cases can only be found by wastewater-based surveillance, which is also important for keeping tabs on problems and alerting relevant parties so that postexposure prophylaxis can be administered [14]. The detection of MPXV at an early stage may be facilitated by monitoring MPXV nucleic acids in sewer water and on surfaces [69]. Testing must be widely available, especially in low and middle-income countries, and a universally recognized case definition for MPX is required. Additionally, it's essential to perform surveillance on animals in close contact with infected patients to prevent the establishment of animal reservoirs [70].

The distribution of information about the dangers of MPX and how to stop it among the general public is also essential. Maintaining proper hygiene practices, such as regular hand hygiene, proper wound or sore inspection, and minimizing contact with wildlife, particularly mice and primates, are crucial preventive strategies [71]. Training health organizations and individuals to identify, isolate, and treat cases of MPX is also crucial, as early detection and immediate treatment can help mitigate the disease's effects and slow its spread. The health and safety of individuals and communities can be safeguarded from the spread of MPX by raising public awareness and encouraging preparation for potential outbreaks [72]. The most effective non-pharmaceutical measure to prevent the spread of MPX is to avoid close contact with infected individuals. Patients and their households should be advised on proper hygiene and disinfection protocols. Surface disinfectants with proven “virucidal activity against enveloped viruses” can be used for disinfection [73].

Controlling any disease requires better laboratory and diagnosis facilities and knowledgeable staff. Because of the potential for infection, a Biosafety Level 3 (BSL-III) laboratory is suggested for work with this virus. Exporting laboratories should strictly adhere to Standard Operating Procedure (SOP) [74]. The use of full PPE (gloves, face shields, goggles, head cover, and foot protection) is required in the lab at all times. Proper waste management calls for planning and adherence to established procedures. It is recommended that those working with infectious materials in laboratories get vaccinated [75].

## 9 Recommendation and conclusion

A rare and infectious MPX can cause mild to life-threatening illness in humans. This brief review article provided an overview of disease, virology, epidemiology, and treatment. The MPXV, an emerging global health concern, may be contained through the cumulative efforts of the governing authorities and the general public, who are responsible for the majority of transmission. A few things can be attributed to the recent multicountry outbreak, such as increased international trade, travel from regions where the virus is endemic to other nations, genetic changes in the virus, unprotective sex with men, ecological factors, wildlife habitats, limited diagnosis resources, a lack of surveillance, and adequate and proper treatment. Human-to-human transmission is a concern not only for family members but also for medical professionals. Academicians in relevant fields should make arduous efforts to investigate novel concepts of preventive measures and therapeutics to protect against outbreaks of emerging variants. To control this current multicountry outbreak, interdepartmental measures must be taken, beginning with the exploration of rapid diagnosis, screening out hotspot areas, and funding additional research for the development of vaccines. It is essential to have tangible measures that can be put into place to effectively address the epidemic if one wants to prevent, reduce, or control the spread of zoonotic diseases. For instance, one may propose the formation of rapid response teams, an increase in the capacity of laboratories, or the distribution of diagnostic kits that are specifically designed for MPX detection. Clinicians must be informed of the current treatment regime and the mode of disease transmission to prevent the disease's further spread by themselves and others. Water-based surveillance needs to be robust for MPXV surveillance in the risk areas, encompassing controls and preventive measures. Expanding lab facilities, open data sharing, following one health rules, developing dashboards, and raising public awareness through social media or other digital channels might also be prioritized. Research-based studies must be carried out to create new antiviral medications, vaccines, and other therapeutic interventions. The implementation of these policies requires the cooperation of many different groups, including government officials, public health organizations, researchers, and healthcare professionals. Although MPXV vaccines can provide immunity against this disease, the lack of vaccines forces the authorities to adopt a different strategy that must include appropriate public health regulations, such as safe sex practices. More MPX-based research is necessary to advance the development of efficient antiviral medicines, vaccines, and therapeutic interventions.

## Appendix Supplementary materials

Image, application 1
