# Supplementary material for: Monkeypox virus (MPXV): A Brief account of global spread, epidemiology, virology, clinical features, pathogenesis, and therapeutic interventions
Source: Infect Med (Beijing). 2023 Nov 5;2(4):262–72. doi: 10.1016/j.imj.2023.11.001 (PMC10774656; doi:10.1016/j.imj.2023.11.001)
Supplement: Supplementary file 1 [file mmc1.docx]

**Monkeypox Virus (MPXV) outbreak: A Brief Account of Global Spread, Epidemiology, Pathogenesis, and Therapeutic Interventions**

**Md Aminul Islam^a,b*^, Md. Azizul Haque^c^, Ahrar Khan^e^, and Md Atiqul Haque^f,g^**

^a^Advanced Molecular Lab, Department of Microbiology, President Abdul Hamid Medical College, Karimganj, Kishoreganj-2310, Bangladesh

^b^COVID-19 Diagnostic lab, Department of Microbiology, Noakhali Science and Technology University, Noakhali-3814, Bangladesh

^c^Department of Biochemistry and Molecular Biology, Faculty of Agriculture, Hajee Mohammad Danesh Science and Technology University, Dinajpur-5200, Bangladesh

^d^COVID-19 Research @KTH, Department of Sustainable Development, Environmental Science and Engineering, KTH Royal Institute

of Technology, Teknikringen 10B, SE-100 44 Stockholm, Sweden

^e^Shandong Vocational Animal Science and Veterinary College, Weifang, 261061, China

^f^Key Laboratory of Animal Epidemiology and Zoonoses of Ministry of Agriculture, College of Veterinary Medicine, China Agricultural University, Beijing 100019, China

^g^Department of Microbiology, Faculty of Veterinary and Animal Science, Hajee Mohammad Danesh Science and Technology University, Dinajpur-5200, Bangladesh

*Corresponding author

**Md. Aminul Islam**

Lecturer, Department of Microbiology

President Abdul Hamid Medical College Hospital, Kishoreganj (PAHMCH)

Senior Research Assistant, NSTU Covid-19 Lab, Department of Microbiology, NSTU

Lab Incharge, Advance Molecular Lab, PAHMCH

E-mail: aminulmbg@gmail.com, [aminul@pahmc.edu.bd](mailto:aminul@pahmc.edu.bd)

**List of Index:**

1. Supplementary file 1: Table S1. Recent outbreak of MPX worldwide January 2022 to 21 Mar 2023 (WHO 2022)

**Table S1. Recent outbreak of MPX worldwide January 2022 to February 2023 (WHO 2022)**

| **S/N** | **Country** | **Total Cases** | Total **Deaths** | **Cases/Million** | **Population** | **First case** |
| --- | --- | --- | --- | --- | --- | --- |
| 1 | USA | 30056 | 38 | 89.52666751 | 334805269 | 18, May |
| 2 | Brazil | 10808 | 15 | 50.18722859 | 215353593 | 15, Jun |
| 3 | Spain | 7546 | 3 | 161.3471412 | 46719142 | 18, May |
| 4 | France | 4128 | 0 | 60.9298893 | 67750000 | n.i. |
| 5 | Colombia | 4088 | 0 | 79.20367384 | 51512762 | 23, Jun |
| 6 | Mexico | 3928 | 4 | 30.21310182 | 126700000 | n.i. |
| 7 | Peru | 3776 | 20 | 111.2692764 | 33720000 | n.i. |
| 8 | UK | 3738 | 0 | 55.47304322 | 67330000 | n.i. |
| 9 | Germany | 3692 | 0 | 44.375 | 83200000 | n.i. |
| 10 | Canada | 1478 | 0 | 38.16993464 | 38250000 | n.i. |
| 11 | Chile | 1437 | 2 | 74.07717169 | 19250195 | 17, Jun |
| 12 | Netherlands | 1262 | 0 | 71.87678266 | 17530000 | n.i. |
| 13 | Argentina | 1120 | 2 | 23.79905305 | 46010234 | 27, May |
| 14 | Italy | 957 | 0 | 15.91363955 | 60262770 | 19, May |
| 15 | Portugal | 951 | 0 | 336.064728 | 2829812 | 29, Jun |
| 16 | Belgium | 793 | 1 | 67.96204204 | 11668278 | 19, May |
| 17 | Nigeria | 820 | 9 | 3.697282099 | 213400000 | n.i. |
| 18 | Switzerland | 552 | 0 | 62.80177764 | 8773637 | 21, May |
| 19 | Ecuador | 524 | 3 | 27.71434854 | 18113361 | 06, Jul |
| 20 | Guatemala | 397 | 0 | 20.01717711 | 18584039 | n.i. |
| 21 | DRC | 439 | 0 | 33.46487138 | 11056370 | 06, Jul |
| 22 | Austria | 327 | 0 | 36.51183564 | 8956000 | n.i. |
| 23 | Bolivia | 265 | 0 | 22.01347225 | 11992656 | n.i. |
| 24 | Israel | 262 | 0 | 27.97949594 | 9364000 | n.i. |
| 25 | Sweden | 260 | 0 | 25.44287483 | 10218971 | 19, May |
| 26 | Ireland | 228 | 0 | 45.41652632 | 5020199 | 27, May |
| 27 | Poland | 215 | 0 | 5.696905798 | 37739785 | 10, Jun |
| 28 | Puerto Rico | 211 | 0 | 64338235.29 | 3.264 | n.i. |
| 29 | Denmark | 196 | 0 | 33.46423083 | 5857000 | n.i. |
| 30 | Australia | 144 | 0 | 5.523846291 | 26068792 | 20, May |
| 31 | Costa Rica | 206 | 1 | 27.16336826 | 5154000 | n.i. |
| 32 | Panama | 189 | 0 | 29.00855505 | 4446964 | 05, Jul |
| 33 | Ghana | 123 | 4 | 3.685653366 | 32830000 | n.i. |
| 34 | Paraguay | 118 | 0 | 13.41392088 | 7305843 | n.i. |
| 35 | Norway | 95 | 0 | 17.2370935 | 5511370 | 31, May |
| 36 | El Salvador | 98 | 0 | 13.43431665 | 6550389 | n.i. |
| 37 | Greece | 87 | 0 | 8.336049819 | 10316637 | 08, Jun |
| 38 | Hungary | 80 | 0 | 8.327903714 | 9606259 | 31, May |
| 39 | Czechia | 71 | 1 | 6.612780885 | 10736784 | 24, May |
| 40 | Luxembourg | 57 | 0 | 88.73376911 | 642371 | 15, Jun |
| 41 | Dominican Republic | 52 | 0 | 4.676258993 | 11120000 | n.i. |
| 42 | Romania | 47 | 0 | 2.469611302 | 19031335 | 13, Jun |
| 43 | Slovenia | 47 | 0 | 22.61753176 | 2078034 | 24, May |
| 44 | Finland | 42 | 0 | 7.560810519 | 5554960 | 27, May |
| 45 | New Zealand | 41 | 0 | 8.00312317 | 5123000 | n.i. |
| 46 | Serbia | 40 | 0 | 4.622665669 | 8653016 | 17, Jun |
| 47 | Croatia | 33 | 0 | 8.12950849 | 4059286 | 23, Jun |
| 48 | Malta | 33 | 0 | 74.31880063 | 444033 | 28, May |
| 49 | Lebanon | 27 | 0 | 4.648667978 | 5593000 | n.i. |
| 50 | CAR | 27 | 1 | 4.03151915 | 5457000 | n.i. |
| 51 | India | 22 | 1 | 0.015640198 | 1406631776 | 14, Jul |
| 52 | Singapore | 21 | 0 | 3.533244296 | 5943546 | 20, Jun |
| 53 | Japan | 59 | 0 | 0.159254893 | 125584838 | 25, Jul |
| 54 | Uruguay | 19 | 0 | 5.434757736 | 3496016 | 29, Jul |
| 55 | Cameroon | 18 | 3 | 0.661764706 | 27200000 | n.i. |
| 56 | Jamaica | 18 | 0 | 6.029960866 | 2985094 | 06, Jul |
| 57 | Sudan | 18 | 1 | 1.549251879 | 11618511 | NI |
| 58 | Honduras | 29 | 0 | 1.565366731 | 10221247 | NI |
| 59 | Iceland | 16 | 0 | 46.32404247 | 345393 | 09, Jun |
| 60 | UAE | 16 | 0 | 1.587020552 | 10081785 | 24, May |
| 61 | Thailand | 17 | 0 | 0.214046584 | 70078203 | 21, Jul |
| 62 | Slovakia | 14 | 0 | 2.564011931 | 5460193 | 07, Jul |
| 63 | Venezuela | 12 | 0 | 0.425531915 | 28200000 | n.i. |
| 64 | Estonia | 11 | 0 | 8.26446281 | 1331000 | n.i. |
| 65 | Bosnia & Herzegovina | 9 | 0 | 2.769812856 | 3249317 | 14, Jul |
| 66 | Cuba | 8 | 1 | 0.707610671 | 11305652 | n.i. |
| 67 | Saudi Arabia | 8 | 0 | 0.222531293 | 35950000 | n.i. |
| 68 | China | 15 | 0 | 0.00481458 | 1453916919 | n.i. |
| 69 | Liberia | 7 | 0 | 1.347968419 | 5193000 | n.i. |
| 70 | Martinique | 7 | 0 | 18.59328517 | 376480 | n.i. |
| 71 | Bulgaria | 6 | 0 | 0.872346612 | 6878000 | n.i. |
| 72 | Gibraltar | 6 | 0 | 178.1948858 | 33671 | 01, Jun |
| 73 | Latvia | 6 | 0 | 3.245283386 | 1848837 | 03, Jun |
| 74 | Uganda | 6 | 0 | 0.123882827 | 48432863 | n.i. |
| 75 | Congo | 5 | 0 | 0.052143081 | 95890000 | n.i. |
| 76 | Cyprus | 5 | 0 | 4.019292605 | 1244000 | n.i. |
| 77 | Lithuania | 5 | 0 | 1.785076758 | 2801000 | n.i. |
| 78 | Qatar | 5 | 0 | 1.677900209 | 2979915 | 22, Jul |
| 79 | South Africa | 5 | 0 | 0.084189257 | 59390000 | n.i. |
| 80 | Ukraine | 5 | 0 | 0.115761851 | 43192122 | n.i. |
| 81 | Andorra | 4 | 0 | 51.63755599 | 77463 | 02, Jul |
| 82 | Philippines | 4 | 0 | 0.035552713 | 112508994 | n.i. |
| 83 | Republic of Korea | 5 | 0 | 77309.62505 | 51.74 | n.i. |
| 84 | Aruba | 3 | 0 | 28.09883296 | 106766 | n.i. |
| 85 | Benin | 3 | 0 | 0.234655009 | 12784726 | 14, Jun |
| 86 | Curacao | 3 | 0 | 18.12425993 | 165524 | 15, Aug |
| 87 | Egypt | 3 | 0 | 0.028260112 | 106156692 | n.i. |
| 88 | Monaco | 3 | 0 | 0.747538542 | 4013171 | n.i. |
| 89 | Morocco | 3 | 0 | 0.079422322 | 37772756 | 02, Jun |
| 90 | Bahamas | 2 | 0 | 4.99355831 | 400516 | n.i. |
| 91 | Georgia | 2 | 0 | 0.503938532 | 3968738 | 15, Jun |
| 92 | Greenland | 2 | 0 | 35.10434767 | 56973 | n.i. |
| 93 | Guyana | 2 | 0 | 2.518748937 | 794045 | 22, Aug |
| 94 | Montenegro | 2 | 0 | 0.592052641 | 3378078 | n.i. |
| 95 | Republic of Moldova | 2 | 0 | 764818.3556 | 2.615 | n.i. |
| 96 | Russian Federation | 2 | 0 | 0.013716862 | 145805947 | 12, Jun |
| 97 | Sri Lanka | 2 | 0 | 0.090252708 | 22160000 | n.i. |
| 98 | Viet Nam | 2 | 0 | 0.020519134 | 97470000 | n.i. |
| 99 | Bahrain | 1 | 0 | 0.560543458 | 1783983 | n.i. |
| 100 | Barbados | 1 | 0 | 3.471944949 | 288023 | 16, Jul |
| 101 | Bermuda | 1 | 0 | 15.78432301 | 63354 | 22, Jul |
| 102 | Guadeloupe | 1 | 0 | 2.501288163 | 399794 | 27, Jul |
| 103 | Guam | 1 | 0 | 5.863933292 | 170534 | n.i. |
| 104 | Indonesia | 1 | 0 | 0.003582502 | 279134505 | n.i. |
| 105 | Iran | 1 | 0 | 0.01162482 | 86022837 | n.i. |
| 106 | Jordan | 1 | 0 | 0.097079188 | 10300869 | n.i. |
| 107 | Mozambique | 1 | 1 | 0.03117207 | 32080000 | n.i. |
| 108 | New Caledonia | 1 | 0 | 3.434313601 | 291179 | n.i. |
| 109 | Saint Martin | 1 | 0 | 24.96442569 | 40057 | 01, Aug |
| 110 | San Marino | 1 | 0 | 29.63401985 | 33745 | n.i. |
| Total | | 86,646 | 112 | - | - | - |

CAR=Central African Republic; DRC=Democratic Republic of the Congo; UAE=United Arab Emirates; UK=United Kingdom; USA=United States of America; n.i.=Not identified

**Reference**

1. World Health Organization (WHO). 2022 Mpox (Monkeypox) Outbreak: Global Trends. https://worldhealthorg.shinyapps.io/mpx_global/#sectionfns2%E2%80%982022%20Monkeypox%20Outbreak:%20Global%20Trends%E2%80%99. Accessed on February 18, 2023.
